# Patients Recovering from Severe COVID-19 Develop a Polyfunctional Antigen-Specific CD4+ T Cell Response

**DOI:** 10.3390/ijms23148004

**Published:** 2022-07-20

**Authors:** Annamaria Paolini, Rebecca Borella, Anita Neroni, Domenico Lo Tartaro, Marco Mattioli, Lucia Fidanza, Alessia Di Nella, Elena Santacroce, Licia Gozzi, Stefano Busani, Tommaso Trenti, Marianna Meschiari, Giovanni Guaraldi, Massimo Girardis, Cristina Mussini, Lara Gibellini, Sara De Biasi, Andrea Cossarizza

**Affiliations:** 1 Department of Medical and Surgical Sciences for Children and Adults, University of Modena and Reggio Emilia School of Medicine, Via Campi 287, 41125 Modena, Italy; annamaria.paolini@unimore.it (A.P.); rebecca.borella@unimore.it (R.B.); anneroni@unimore.it (A.N.); domenico.lotartaro@unimore.it (D.L.T.); mattioli.marco@gmail.com (M.M.); lucia.fidanza@unimore.it (L.F.); adinella@unimore.it (A.D.N.); 239216@studenti.unimore.it (E.S.); lara.gibellini@unimore.it (L.G.); andrea.cossarizza@unimore.it (A.C.); 2 Infectious Diseases Clinics, AOU Policlinico di Modena, Via del Pozzo 71, 41124 Modena, Italy; licia.gozzi@gmail.com (L.G.); mariannameschiari1209@gmail.com (M.M.); giovanni.guaraldi@unimore.it (G.G.); cristina.mussini@unimore.it (C.M.); 3 Department of Surgery, Medicine, Dentistry and Morphological Sciences, University of Modena and Reggio Emilia, Via del Pozzo 71, 41124 Modena, Italy; stefano.busani@unimore.it (S.B.); massimo.girardis@unimore.it (M.G.); 4 Department of Anesthesia and Intensive Care, AOU Policlinico and University of Modena and Reggio Emilia, Via del Pozzo 71, 41124 Modena, Italy; 5 Department of Laboratory Medicine and Pathology, Diagnostic Hematology and Clinical Genomics, AUSL/AOU Policlinico, 41124 Modena, Italy; t.trenti@ausl.mo.it; 6 National Institute for Cardiovascular Research, Via Irnerio 48, 40126 Bologna, Italy

**Keywords:** COVID-19, antigen-specific T cells, cytokine production, polyfunctionality, flow cytometry

## Abstract

Specific T cells are crucial to control SARS-CoV-2 infection, avoid reinfection and confer protection after vaccination. We have studied patients with severe or moderate COVID-19 pneumonia, compared to patients who recovered from a severe or moderate infection that had occurred about 4 months before the analyses. In all these subjects, we assessed the polyfunctionality of virus-specific CD4+ and CD8+ T cells by quantifying cytokine production after in vitro stimulation with different SARS-CoV-2 peptide pools covering different proteins (M, N and S). In particular, we quantified the percentage of CD4+ and CD8+ T cells simultaneously producing interferon-γ, tumor necrosis factor, interleukin (IL)-2, IL-17, granzyme B, and expressing CD107a. Recovered patients who experienced a severe disease display high proportions of antigen-specific CD4+ T cells producing Th1 and Th17 cytokines and are characterized by polyfunctional SARS-CoV-2-specific CD4+ T cells. A similar profile was found in patients experiencing a moderate form of COVID-19 pneumonia. No main differences in polyfunctionality were observed among the CD8+ T cell compartments, even if the proportion of responding cells was higher during the infection. The identification of those functional cell subsets that might influence protection can thus help in better understanding the complexity of immune response to SARS-CoV-2.

## 1. Introduction

The characterization of the immune response mounted against Severe Acute Respiratory Syndrome-Coronavirus-2 (SARS-CoV-2) infection is crucial to understanding and predicting short- and long-term protection. Both innate and adaptive immunity has been well described during severe cases as well as in recovered patients [1,2,3,4,5,6,7,8,9,10] and it has been reported that an integrated response can limit COVID-19 disease severity [11]. Developing SARS-CoV-2 antigen-specific CD4+ and CD8+ T cells besides antibodies is crucial to prevent severe outcomes and protect against reinfections [11,12]. This explains, at least in part, why: (i) immunocompromised patients with reduced humoral response and deficient B cells can develop a SARS-CoV-2 specific T cell response [13]; (ii) patients experiencing mild COVID-19 can successfully control the virus thanks to a robust SARS-CoV-2 T cell response even in the absence of antibodies [11,14,15,16,17].

SARS-CoV-2 T cell response in patients recovered from COVID-19 is multi-specific as T cells recognize several epitopes, by using a heterogenous T cell receptor (TCR) [18,19,20,21]. Functional studies using peptide pools covering most of SARS-CoV-2 encoded proteome demonstrated that T cell response to structural proteins such as the membrane (M), spike (S) or nucleocapsid (N) is co-dominant and that a significant reactivity is also developed against other targets, such as Open Reading Frames (ORFs) and nonstructural proteins (NSPs) [5,18,19]. However, whether this multi-specificity is the key to long-term protection is still uncertain.

CD4+ and CD8+ T cell polyfunctionality indicate the ability of cells to simultaneously produce more than one cytokine and to exert multiple functions. This is a crucial feature in antigen-specific responses as, in some cases, the quality of the response can be more important than the quantity in conferring protection against reinfection or pathogen reactivation [22,23]. In this scenario, CD4+ T helper type 1 (Th1) and Th17 are fundamental in inducing CD8+ T and B cells activity and promoting a pro-inflammatory response [12,24,25]. For example, Th1 and Th17 CD4+ T and CD8+ T cells dominate the influenza A virus-specific response, so inducing both a highly inflammatory environment and viral clearance [26,27,28].

For these reasons, given the role and capability of these cells, the aim of the study is to characterize the polyfunctional profile of SARS-CoV-2-specific T cells. Moreover, we aimed to investigate possible differences in the specific response between patients experiencing and recovering from moderate or severe infection, deepening at the same time the immunogenic capacity of M, N and S SARS-CoV-2 structural proteins.

## 2. Results

### 2.1. Characteristics of the Patients

We studied a total of 28 patients with COVID-19 pneumonia admitted into the Infectious Diseases Clinics or to the Intensive Care Unit (ICU) of the University Hospital in Modena over the period of March 2020–May 2020, and 10 healthy donors.

Characteristics of patients are reported in Table 1. COVID-19 moderate and COVID-severe presented higher levels of LDH when compared to recovered moderate and recovered severe, respectively. Regarding SARS-CoV-2-specific IgM and IgG, even if IgM were more represented among patients with moderate disease, no statistically significant differences were found between those with COVID-19 and the recovered, while HD tested negative for both assays. One patient from the COVID-19 severe group and one from the recovered severe group presented with type 2 diabetes. Recovered moderate and recovered severe were hospitalized and diagnosed with SARS-CoV-2 infection 120 ± 18 (mean ± SD) days and 128 ± 3 (mean ± SD) days, respectively, before blood withdrawal.

An example of the gating strategy for the identification of cells able to exert one or more functions is reported in Figure S1. Peripheral blood mononuclear cells (PBMCs) were stimulated or not with M, N or S peptide pool, cultured and stained. PBMCs were first gated according to their physical parameters, and the aggregates were electronically removed from the analysis by using a gate designed for singlets. Living (Live/Dead, L/D-) cells and CD3+ T cells were identified. Among CD3+ cells, CD4+ and CD8+ T cell subpopulations were identified. In each subpopulation, the percentage of cells producing interferon (IFN)-γ, Tumor Necrosis Factor (TNF), Interleukin (IL)-2, IL-17, and granzyme B (GRZB), as well as expression of CD107a, was then quantified.

### 2.2. Recovered Patients Who Experienced a Severe Disease Display High Percentage of Antigen-Specific CD4+ T Cells Producing Th1 and Th17 Cytokines

Cytokine production was assessed following 16 h of in vitro stimulation with SARS-CoV-2 peptide pools covering the sequence of different proteins (N, M or S). The percentage of CD4+ and CD8+ T cells producing IFN-γ, TNF, IL-2, IL-17, and GRZB was quantified along with the percentage of cells able to express CD107a. The identification of these cytokines allows us to recognize different subsets of helper CD4+ and CD8+ T cells, such as: (i) Th1, defined as cells producing IFN-γ, TNF, IL-2; (ii) Th17 identified as cells producing IL-17; (iii) cytotoxic T cells, which are positive for GRZB and CD107a [29,30].

**Figure 1:**
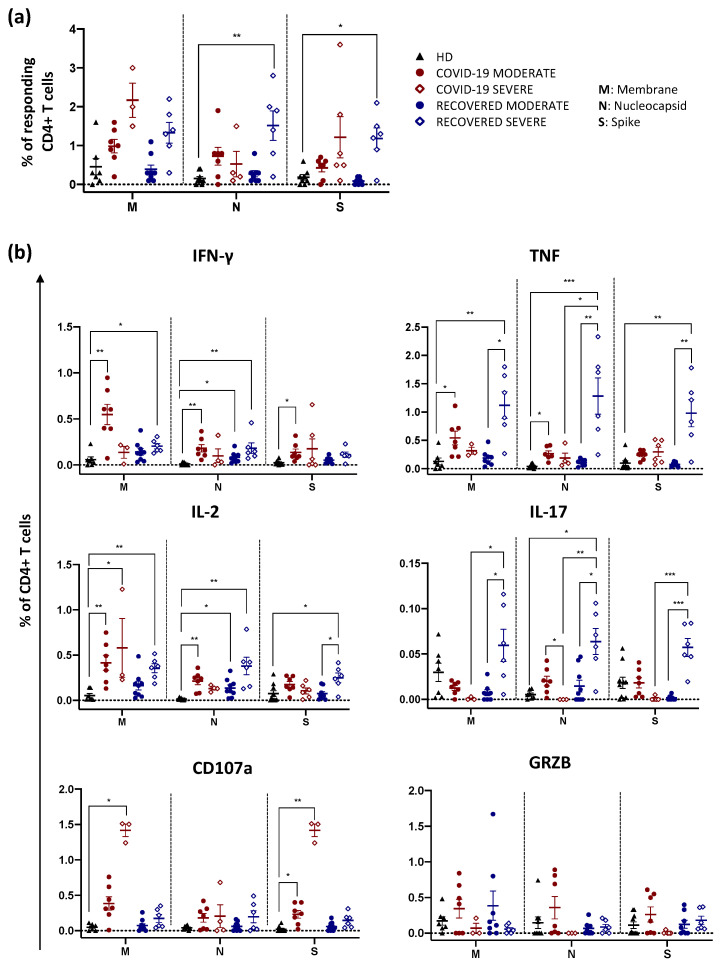
Total cytokine production by CD4+ T cells after in vitro stimulation. (**a**) Percentage of responding CD4+ T cells after stimulation with M, N or S. Data represent individual values from healthy donors (HD, *n* = 10), COVID-19 moderate (*n* = 7), COVID-19 severe (*n* = 6), recovered moderate (*n* = 9) and recovered severe (*n* = 6). Mean (center bar) ± standard error of the mean (SEM, upper and lower bars) is represented. Statistical analysis was performed using Kruskal–Wallis non-parametric test corrected for multiple comparisons by controlling the False Discovery Rate (FDR), method of Benjamini and Hochberg. * *q* < 0.05; ** *q* < 0.01. Background was subtracted from each sample. (**b**) Representation of the total production of each cytokine after stimulation of CD4+ T cells. We evaluated the percentage of CD4+ T cells producing IFN-γ, TNF, IL-2, IL-17, GRZB as well as expressing CD107a among HD (*n* = 10), COVID-19 moderate (*n* = 7), COVID-19 severe (*n* = 6), recovered moderate (*n* = 9) and recovered severe (*n* = 6). Data are represented as individual values, mean (center bar) ± standard error of the mean (SEM, upper and lower bars) is represented. Statistical analysis was performed using Kruskal–Wallis non-parametric test corrected for multiple comparisons by controlling the False Discovery Rate (FDR), method of Benjamini and Hochberg. * *q* < 0.05; ** *q* < 0.01; *** *q* < 0.001. Background (i.e., the value determined in unstimulated controls) was subtracted from each sample.

Individuals who recovered from a severe form of COVID-19 disease showed a higher percentage of CD4+ T cells responding to N and S compared to healthy donors (HD) (Figure 1a). Moreover, taking into consideration all the stimuli used, patients who recovered from a severe disease exhibited a higher percentage of CD4+ T cells producing IFN-γ, TNF and IL-2 compared to either HD or individuals who recovered from moderate disease (Figure 1b). This was also observed when COVID-19 patients with a moderate disease were compared to HD. Furthermore, recovered individuals who experienced a severe disease also displayed a higher percentage of CD4+ T cells producing IL-17 compared to recovered moderate, regardless of the stimulus used (Figure 1b). On the other hand, COVID-19 patients with severe infection were characterized by higher proportions of cells expressing CD107a compared to HD after M and S stimulation, indicating a more enhanced cytotoxic phenotype (Figure 1b).

**Figure 2:**
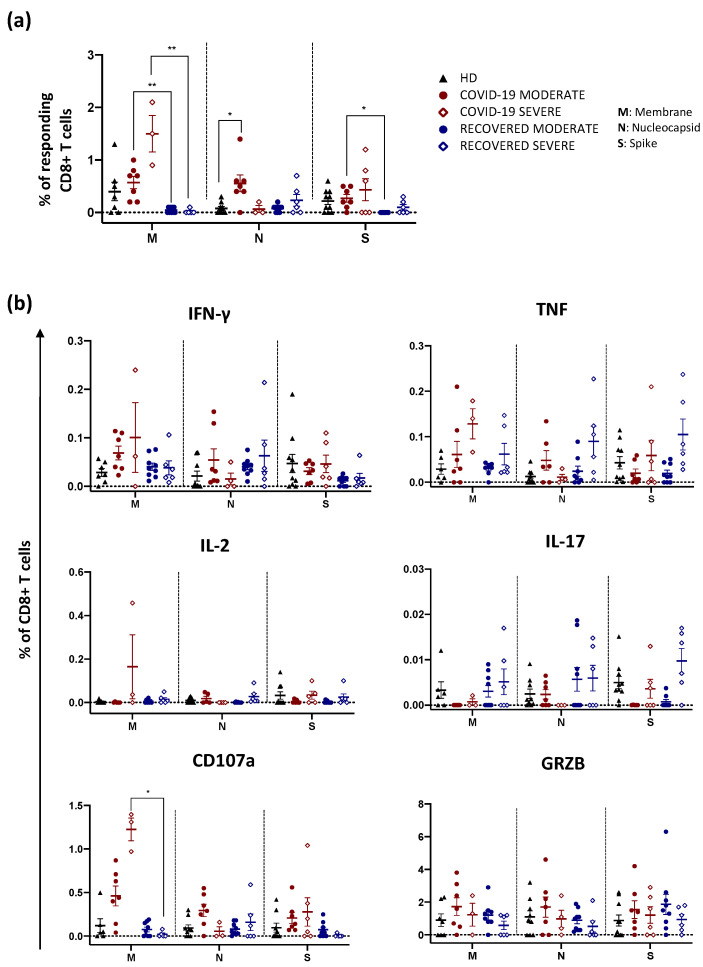
Total cytokine production by CD8+ T cells after in vitro stimulation. (**a**) Percentage of responding CD8+ T cells after stimulation with M, N or S. Data represent individual values from HD (*n* = 10), COVID-19 moderate (*n* = 7), COVID-19 severe (*n* = 6), recovered moderate (*n* = 9) and recovered severe (*n* = 6). Mean (center bar) ± standard error of the mean (SEM, upper and lower bars) is represented. Statistical analysis was performed using Kruskal–Wallis non-parametric test corrected for multiple comparisons by controlling the False Discovery Rate (FDR), method of Benjamini and Hochberg. * *q* < 0.05; ** *q* < 0.01. Background was subtracted from each sample. (**b**) Representation of the total production of cytokines after stimulation of CD8+ T cells. We evaluated the percentage of CD8+ T cells producing IFN-γ, TNF, IL-2, IL-17, GRZB as well as expressing CD107a among HD (*n* = 10), COVID-19 moderate (*n* = 7), COVID-19 severe (*n* = 6), recovered moderate (*n* = 9) and recovered severe (*n* = 6). Data are represented as individual values, mean (center bar) ± standard error of the mean (SEM, upper and lower bars) is represented. Statistical analysis was performed using Kruskal–Wallis non-parametric test corrected for multiple comparisons by controlling the False Discovery Rate (FDR), method of Benjamini and Hochberg. * *q* < 0.05. Background (i.e., the value determined in unstimulated controls) was subtracted from each sample.

Regarding CD8+ T cell response, the percentage of CD8+ T cells responding to peptide pool stimulation was higher in COVID-19 patients with a moderate disease compared to either HD or recovered individuals who experienced a moderate infection. In addition, COVID-19 patients with severe form exhibited a higher percentage of responding CD8+ T cells compared to those who recovered from a severe form (Figure 2a). Furthermore, after in vitro stimulation with M, COVID-19 severe patients displayed a higher percentage of CD8+ T cells expressing CD107a compared to individuals who recovered from severe infection (Figure 2b). Thus, antigen-specific CD8+ T cells are more abundant among COVID-19 patients and present a more pronounced cytotoxic phenotype in line with their role in mediating clearance during viral infections [31].

### 2.3. Recovered Patients Who Experienced a Severe Disease Are Characterized by Polyfunctional SARS-CoV-2 Antigen-Specific CD4+ T cells

**Figure 3:**
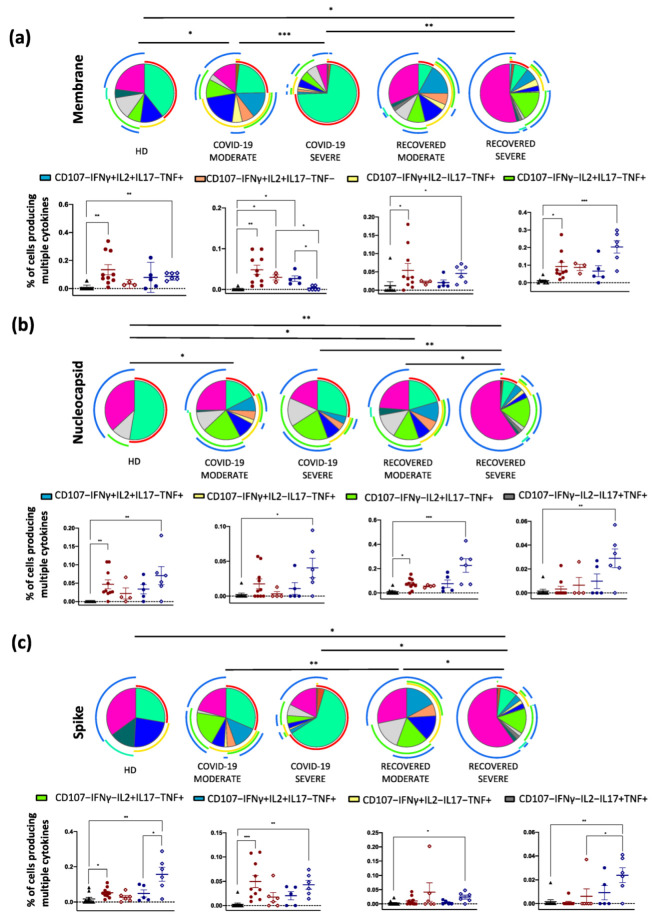

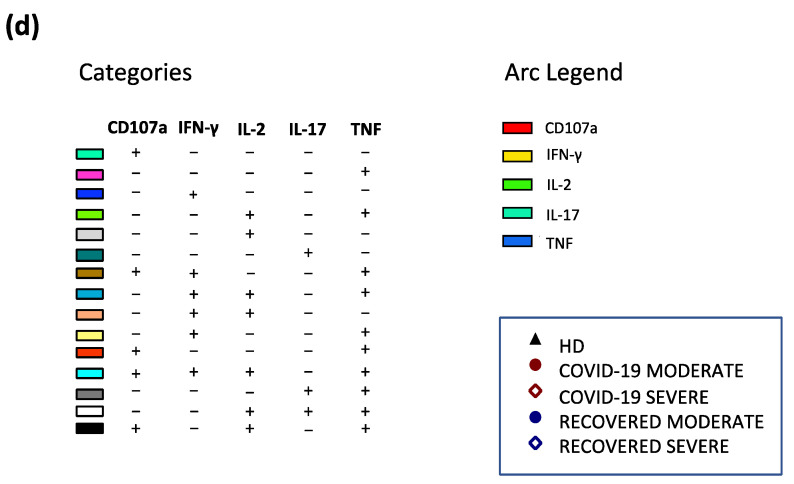
Polyfunctionality of CD4+ T cells after in vitro stimulation. Pie charts representing the proportion of CD4+ T cells producing different combinations of IFN-γ TNF, IL-2, IL-17, GRZB as well as expressing CD107a after stimulation with (**a**) M; (**b**) N; or (**c**) S peptide pools from HD (*n* = 10), COVID-19 moderate (*n* = 7), COVID-19 severe (*n* = 6), recovered moderate (*n* = 9) and recovered severe (*n* = 6) patients. For clarity, panel (**d**) reports the legend for the colors and the symbols used in panels (**a**–**c**). Data in pie charts are represented as median values. Frequencies were corrected by background subtraction as determined in non-stimulated controls using SPICE software. Statistical analysis between pie charts was performed using permutation test (* *p* < 0.05; ** *p* < 0.01; *** *p* < 0.001). Pie arches represent the total production of different cytokines. Comparison between the production of different combinations of cytokines by CD4+ T cells is represented. Data are represented as individual values, mean (center bar) ± standard error of the mean (SEM, upper and lower bars) is represented. Statistical analysis was performed using Kruskal–Wallis non-parametric test corrected for multiple comparisons by controlling the False Discovery Rate (FDR), method of Benjamini and Hochberg. * *q* < 0.05; ** *q* < 0.01; *** *q* < 0.001.

In vitro stimulation with the M peptide pool induced a different polyfunctional profile between COVID-19 moderate and severe patients, COVID-19 severe patients and those who recovered from severe disease. Moreover, the polyfunctional response was different when compared to HD in either patients with moderate COVID-19 or those who recovered from severe disease. In particular, COVID-19 moderate patients and recovered individuals from severe disease, when compared to HD, reported higher percentages of IFN-γ+IL-2+TNF+, IFN-γ+TNF+ and IL-2+TNF+ within CD4+ T cells. Patients experiencing COVID-19 moderate also displayed a high percentage of IFN-γ+IL-2+ within CD4+ T cells. The percentage of the latest population was higher in COVID-19 severe and recovered moderate if compared to recovered severe and HD (Figure 3a).

Stimulation with N induced differences in the overall polyfunctionality of CD4+ T cells between patients who recovered (moderate vs. severe) and between COVID-19 severe patients and those who recovered from severe disease. Finally, COVID-19 moderate patients and recovered displayed a different cytokine profile when compared to HD. Regarding the subsets of polyfunctional CD4+ T cells, individuals who recovered from the severe disease exhibited the same cytokine production as seen with M stimulation. In addition, this group of patients presented a small population of TNF+, IL-17+ cells. COVID-19 moderate patients, compared to HD, also presented a high percentage of IFN-γ+IL-2+TNF+ and IL-2+TNF+ (Figure 3b).

Finally, after stimulation with S, individuals who recovered from different disease severity showed a different polyfunctionality as well as COVID-19 moderate patients and recovered from the moderate disease. In addition, recovered from severe disease displayed a distinct polyfunctional asset compared to COVID-19 severe and HD. Individuals who recovered from severe disease presented almost overlapping results as those observed after stimulation with N and M. Moreover, they also displayed a higher percentage of TNF+IL-17+ within CD4+ T cells if compared to COVID-19 severe and HD. Regarding COVID-19 moderate, the cell distribution after stimulation is the same as the one measured after N stimulation (Figure 3c). For clarity, Figure 3d shows the legend of the colors and symbols of the previous Figure 3 panels.

**Figure 4:**
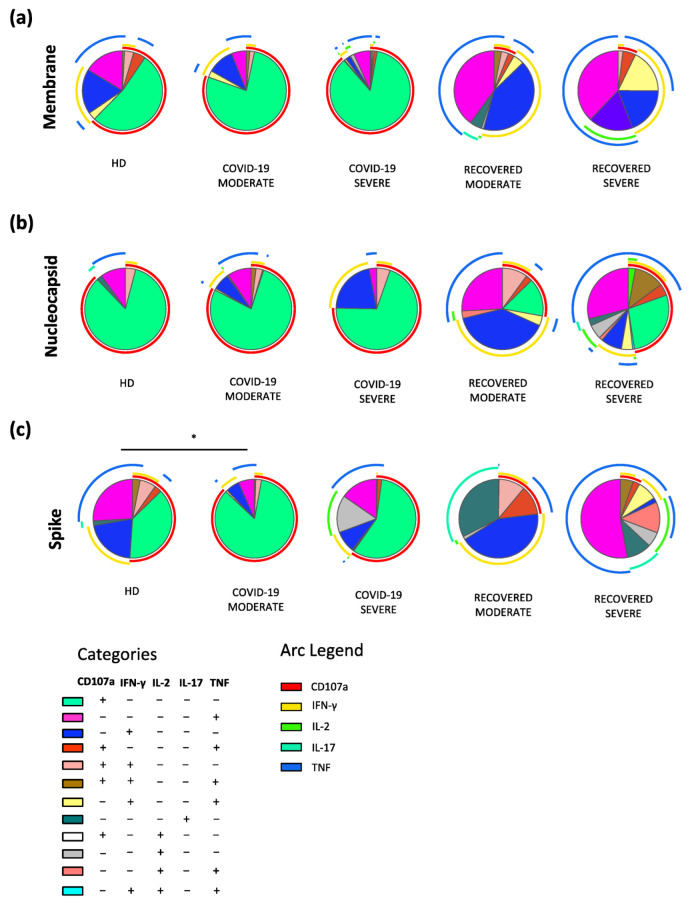
Polyfunctionality of CD8+ T cells after in vitro stimulation. Pie charts representing the proportion of CD8+ T cells producing different combinations of IFN- γ, TNF, IL-2, IL-17, GRZB as well as expressing CD107a after stimulation with (**a**) M; (**b**) N; or (**c**) S peptide pools from HD (*n* = 10), COVID-19 moderate (*n* = 7), COVID-19 severe (*n* = 6), recovered moderate (*n* = 9) and recovered severe (*n* = 6) patients. Data in pie charts are represented as median values. Frequencies were corrected by background subtraction as determined in non-stimulated controls using SPICE software. Statistical analysis between pie charts was performed using permutation test (* *p* < 0.05). Pie arches represent the total production of different cytokines.

The polyfunctional profile of CD8+ T cells after in vitro stimulation with M or N was similar among the groups. Only the S peptide pool induced a slightly different profile in COVID-19 moderate patients when compared to HD (Figure 4).

## 3. Discussion

In this study, we describe the differences in the production of cytokines by SARS-CoV-2-specific T cells from patients with COVID-19 (severe or moderate) and in recovered individuals after in vitro stimulation with different peptide pools. Our aim was to measure not only the magnitude but also the characteristics, in qualitative terms, of such antigen-specific response. We found that COVID-19 moderate patients develop polyfunctional CD4+ T cells compared to patients experiencing a severe infection, that in turn display a higher percentage of CD107a+ cells. Besides their helper capability, CD4+ T cells can exert cytotoxicity, and this has been described during persistent infections such as those by Epstein–Barr virus [32], cytomegalovirus [33], and Human Immunodeficiency Virus (HIV) [34]. Cytotoxic potential can be measured, detecting the expression of the degranulation marker CD107a [35]. This result is in line with other studies demonstrating that patients experiencing severe COVID-19 usually mount an impaired SARS-CoV-2 T cell-specific response [11,36]. It is known that the expression of exhaustion markers such as Programmed Death-1 (PD-1) and T-cell immunoglobulin and mucin domain-3 (Tim-3) is associated with disease progression [37,38]. This might reinforce the concept that patients experiencing a more severe infection present impaired CD4+ and CD8+ T cell functionality due to an exhausted phenotype. However, whether the expression of such markers reflects functional exhaustion rather than ongoing activation is still debated [37].

During the infection, Th1 cytokines such as IFN-γ, IL-2 and TNF are essential for supporting the expansion and maturation of CD8+ T lymphocytes and B cells [12]. The loss of CD4+ Th1 leads to a progressive CD8+ T cell decline and dysfunction with important implications for controlling the infection [39]. In addition, Th17 cells are responsible for the recruitment of several different cell populations at the site of the infection, inducing the inflammatory process necessary for the immediate protective response against a pathogen [24,25]. We found that, if compared to patients experiencing severe COVID-19, those recovering from severe COVID-19 display SARS-CoV-2-specific, highly polyfunctional CD4+ T cells with a Th1 and Th17 phenotype. No differences were reported in the CD8+ T cell compartment, reflecting the T cell kinetics of the immune response contraction according to which 2 weeks after onset symptoms, when circulating CD8+ T cells progressively decline, CD4+ T cells remain stable and eventually increase in the initial recovery phase (1–2 months after infection), more than immediately after infection [11,40].

T cells are able to both proliferate and secrete cytokines that in turn can influence other cell functions as well as induce cytolysis of infected cells. Polyfunctionality is the ability of cells to simultaneously perform more than one function, and it can be measured at a single cell level by flow cytometry [41]. In CD4+ T cells, such property is a correlate for protection against different pathogens. As an example, comparing the profile (more than the amount) of T cell cytokine production in different groups of HIV-infected individuals such as in those who control the infection to that of patients with a chronic progression of the infection revealed the presence of several key molecules involved in controlling the infection. This approach suggested that in some cases the quality of the T cell response, not the quantity, is correlated with immune protection [23]. During cytomegalovirus (CMV) infection, the development of polyfunctional T cells correlates with a better prognosis and confers an immunological advantage against other pathogens [22]. In addition, polyfunctional CD4+ T cells represent a marker for spontaneous control of viral replication in CMV-seropositive patients undergoing liver transplantation [42]. On the whole, this indicates the importance of measuring representative functions of T cells to identify and define correlates of immune protection.

The identification of the most immunogenic epitopes is key to the study and understanding of cellular immune response to gain insights into virus-induced infection mechanisms. An immunogenic peptide is one that is presented by a self-major histocompatibility complex (MHC) and is able to elicit a T cell response [43]. Thus, the identification of such epitopes is also of importance in the context of future therapies. M, N and S are SARS-CoV-2 structural proteins that constitute different portions of the virus. These proteins have different interactions with the other parts of the virion, and during the infection, they interact differently and in different moments with the host cell. This may define a different level of immunogenicity for each protein. For these reasons, we deepened the SARS-CoV-2 specific response to M, N and S. Overall, in our study we observed that M, N and S induced a similar response among the categories considered, confirming their co-dominance [18,44].

We are aware that this study has a main limitation since the number of individuals that we could study is relatively small, because of the difficulties to obtain biological material from patients admitted to the hospital. However, even if we could study a relatively low number of patients, we could define the polyfunctionality profile of CD4+ and CD8+ T cells during and after SARS-CoV-2 infection in patients experiencing different severities of COVID-19. Global knowledge of the complex interaction during the cellular response to infection, as well as SARS-CoV-2-induced changes, is helping in understanding mechanisms beyond the immune response toward protective phenotype. In addition, the identification of unique cell subsets involved in immune protection could allow us to develop and use more and more sophisticated techniques that accurately measure the outcome of new therapies. Thus, the successful use of functional T cell analyses will likely help to significantly advance the field of SARS-CoV-2 therapy as well as vaccine efficacy, and hopefully, aid in reducing the global burden of the pandemic.

## 4. Materials and Methods

### 4.1. Patients

Four groups of patients were enrolled in this study, along with a group of healthy donors (HD). We enrolled 13 COVID-19 patients admitted into the Infectious Diseases Clinics or Intensive Care Unit (ICU) of the University Hospital in Modena between March and May 2020. Patients tested positive for the SARS-CoV-2 PCR test. Within this group, 7 patients (median age: 55.0 years) were classified as moderate and 6 (63.0 years) as severe, according to World Health Organization guidelines [45]. We also studied 15 COVID-19 recovered patients, enrolled during follow-up visits between June and August 2020. Within this group, 9 patients (56.0 years) were classified as moderate and 6 (56.5 years) as severe. COVID-19 and recovered patients were subdivided for the analysis according to disease severity. Moreover, 10 HD (49.5 years) were included in this study. HD presented neither symptoms nor prior diagnosis of SARS-CoV-2 and had negative serology. Informed consent, according to Helsinki Declaration, was provided by each participant. All uses of human material have been approved by the local Ethical Committee (Comitato Etico dell’Area Vasta Emilia Nord, protocol number 177/2020, 11 March 2020) and by the University Hospital Committee (Direzione Sanitaria dell’Azienda Ospedaliero-Universitaria di Modena, protocol number 7531, 11 March 2020).

### 4.2. Blood Processing

Blood samples were obtained after informed consent. For COVID-19 patients, blood was obtained after diagnosis of SARS-CoV-2 infection during hospitalization. For recovered patients, blood was collected during a follow-up visit within 120–128 days after hospital admission and SARS-CoV-2 diagnosis. Up to 20 mL of blood were collected from each patient in vacuettes containing ethylenediamine-tetraacetic acid. Peripheral blood mononuclear cells (PBMCs) were isolated according to standard procedures and stored in liquid nitrogen until use [46].

Plasma was collected and stored at −80 °C until the quantification of IgM and IgG, performed according to standard methods by SARS-CoV-2 IgM or IgG Quant Reagent Kit for use with Alinity (Abbott, Abbott Park, IL, USA).

### 4.3. In Vitro Stimulation and Intracellular Cytokine Staining (ICS)

For functional assays on cytokine production by T cells, isolated PBMCs were thawed and rested for 6 h. PBMCs were cultured in the presence of 15-mer peptides with 11-amino acids overlap, covering the sequence of different proteins of SARS-CoV-2: Nucleocapsid phosphoprotein (“N”) (PepTivator SARS-CoV-2 Prot_N), Membrane glycoprotein (“M”) (PepTivator SARS-CoV-2 Prot_M) and Spike glycoprotein (PepTivator SARS-CoV-2 Prot_S) (Miltenyi Biotec, Bergisch Gladbach, Germany). Each peptide was tested separately and 1 μg/mL of anti-CD28 (Miltenyi Biotec, Bergisch Gladbach, Germany) was added to each condition. PBMCs were stimulated for 16 h at 37 °C in a 5% CO_2_ atmosphere in a complete culture medium (RPMI 1640 supplemented with 10% fetal bovine serum and 1% each of l-glutamine, sodium pyruvate, nonessential amino acids, antibiotics, 0.1 M HEPES, 55 μM β-mercaptoethanol). A negative control with unstimulated cells was included in the experimental conditions. All samples were incubated with a protein transport inhibitor containing brefeldin A (Golgi Plug, BD Biosciences Pharmingen, San Diego, CA, USA), a protein transport inhibitor containing monensin (Golgi Stop, BD Biosciences Pharmingen, San Diego, CA, USA) and mAb CD107a-PE (Biolegend, San Diego, CA, USA) at a previously defined concentration. After stimulation, cells were stained with LIVE-DEAD Aqua (ThermoFisher Scientific, Eugene, OR, USA) and surface mAbs recognizing CD3 PE-Cy5, CD4 AF700, and CD8 APC-Cy7 (Biolegend, San Diego, CA, USA). Cells were washed with stain buffer, fixed and permeabilized with the Cytofix/Cytoperm buffer set (BD Biosciences Pharmingen, San Diego, CA, USA) for cytokine detection [47]. Cells were stained with previously titrated directly conjugated mAbs: IL-17A-PE-Cy7, TNF-BV605, IFN-γ-FITC, IL-2-APC and GRZB-BV421 (all mAbs from Biolegend, San Diego, CA, USA). Cells were analyzed by an Attune NxT acoustic cytometer (ThermoFisher Scientific, Eugene, OR, USA). Table S1 reports mAb titers, producer, clone, catalog number, lot number and type of fluorochrome used in the panel.

### 4.4. Statistical Analysis

Quantitative variables were compared using the Kruskal–Wallis non-parametric test corrected for multiple comparisons by controlling the False Discovery Rate (FDR), method of Benjamini and Hochberg. Statistically significant *q* values are represented (* *q* < 0.05; ** *q* < 0.01; *** *q* < 0.001). T cell polyfunctionality was defined by using Simplified Presentation of Incredibly Complex Evaluation (SPICE) software (version 6, kindly provided by Dr. Mario Roederer, Vaccine Research Center, NIAID, NIH, Bethesda, MD, USA) [48]. Data from the total cytokine production are represented as individual values, means, and standard errors of the mean. Regarding polyfunctionality, data in pie charts are represented as median values; statistical analysis was performed using permutation test (* *p* < 0.05; ** *p* < 0.01; *** *p* < 0.001). Data in graphs are reported as individual values, means and standard errors of the mean. Statistical analyses were carried out using Prism 6.0 (GraphPad Software Inc., La Jolla, CA, USA). Background was subtracted from each sample.

## Figures and Tables

**Table 1:** Demographic and clinical characteristics of healthy donors (HD), COVID-19 and recovered patients.

Variable	Healthy Donor(*n* = 10)	COVID-19 Moderate (*n* = 7)	COVID-19 Severe (*n* = 6)	Recovered Moderate (*n* = 9)	Recovered Severe (*n* = 6)	*p*-Value COVID-19 Moderate vs. COVID-19 Severe	*p*-Value COVID-19 Moderate vs. Recovered Moderate	*p*-Value Recovered Moderate vs. Recovered Severe	*p*-Value COVID-19 Severe vs. Recovered Severe
**Demographic** **characteristics**									
Age (median years, range) ^1^	49.5 (37–70)	55.0 (43–65)	63.0 (53–68)	56.0 (36–63)	56.5 (43–61)	ns	ns	ns	ns
Sex (M, %) ^2^	5 (50)	6 (85.7)	6 (100)	5 (55.6)	4 (66.7)	ns	ns	ns	ns
**Clinical** **characteristics**									
*Coexisting conditions*									
Type 2 diabetes, N (%) ^2^	/	0 (0)	1 (16.7)	0 (0)	1 (16.7)	ns	ns	ns	ns
Cardiovascular Dis., N (%) ^2^	/	0 (0)	0 (0)	0 (0)	0 (0)	NA	NA	NA	NA
Chronic Kidney Dis., N (%) ^2^	/	0 (0)	0 (0)	0 (0)	0 (0)	NA	NA	NA	NA
Cancer., N (%) ^2^	/	0 (0)	0 (0)	0 (0)	0 (0)	NA	NA	NA	NA
**Clinical Blood parameters**									
Total bilirubin, mg/dL (median, range) ^1^	/	1.0(0.6–1.4)	0.8(0.3–0.9)	0.7(0.3–0.9)	0.4(0.3–0.8)	ns	ns	ns	ns
CK, U/L (median, range) ^1^	/	81.0 (56.0–154.0)	34.5 (23.0–259.0)	102.0 (87.0–139.0)	139.0 (12.0–282.0)	ns	ns	ns	ns
Creatinine, mg/dL (median, range) ^1^	/	0.8 (0.6–1.0)	0.6 (0.5–0.8)	0.9 (0.7–1.1)	0.9 (0.8–1.2)	ns	ns	ns	ns
D-dimer, ng/mL (median, range) ^1^	/	495 (230–7810)	750 (190–5820)	180 (100.0–340.0)	255 (140.0–780.0)	ns	ns	ns	ns
LDH, U/L (median, range) ^1^	/	591 (580–886)	581 (507.0–1521)	361 (244–450)	384 (337–430)	ns	0.0272	ns	0.0272
CRP, mg/dL (median, range) ^1^	/	0.3 (0.2–0.9)	0.35 (0.2–12.1)	0.2 (0.2–0.3)	0.2 (0.2–0.4)	ns	ns	ns	ns
**Blood cell count**									
White blood cells, N/μL (median, range) ^1^	/	7500 (2888–10,880)	6305 (4800–15,300)	6480 (4420.0–7160)	6985 (6340.0–7680)	ns	ns	ns	ns
Lymphocytes, N/μL (median, range) ^1^	/	2898 (2698–3098)	1642 (629–2460)	2240 (1600–7160)	2615 (2120–3740)	ns	ns	ns	ns
Neutrophils, N/μL (median, range) ^1^	/	6390 (5545–7235)	3818 (1906–14,560)	3120 (2430–2980)	3755 (3060.0–3900)	ns	ns	ns	ns
**Detection of SARS-CoV-2 IgM, IgG**									
IgM, Index (median, range) ^1^	0.0	28.7 (5.7–59.1)	6.4 (1.2–66.3)	4.7 (0.3–28.6)	4.5 (0.5–20.0)	ns	ns	ns	ns
IgG, Index (median, range) ^1^	0.0	7.3 (6.3–9.0)	6.2 (1.5–8.6)	6.1 (2.0–9.4)	3.9(1.2–7.0)	ns	ns	ns	ns

NA; not applicable. ns; not significant, *p*-Value > 0.05. ^1^ Kruskal–Wallis test with original FDR method of Benjamini and Hochberg. ^2^ Chi-square test.

## Data Availability

All flow cytometry data files are available on request.

## Supplementary Materials

The following supporting information can be downloaded at: https://www.mdpi.com/article/10.3390/ijms23148004/s1.

## References

[1] De Biasi S., Meschiari M., Gibellini L., Bellinazzi C., Borella R., Fidanza L., Gozzi L., Iannone A., Lo Tartaro D., Mattioli M. (2020). Marked T cell activation, senescence, exhaustion and skewing towards TH17 in patients with COVID-19 pneumonia. Nat. Commun..

[2] De Biasi S., Lo Tartaro D., Meschiari M., Gibellini L., Bellinazzi C., Borella R., Fidanza L., Mattioli M., Paolini A., Gozzi L. (2020). Expansion of plasmablasts and loss of memory B cells in peripheral blood from COVID-19 patients with pneumonia. Eur. J. Immunol..

[3] Gibellini L., De Biasi S., Paolini A., Borella R., Boraldi F., Mattioli M., Lo Tartaro D., Fidanza L., Caro-Maldonado A., Meschiari M. (2020). Altered bioenergetics and mitochondrial dysfunction of monocytes in patients with COVID-19 pneumonia. EMBO Mol. Med..

[4] Dan J.M., Mateus J., Kato Y., Hastie K.M., Yu E.D., Faliti C.E., Grifoni A., Ramirez S.I., Haupt S., Frazier A. (2021). Immunological memory to SARS-CoV-2 assessed for up to 8 months after infection. Science.

[5] Gangaev A., Ketelaars S.L.C., Isaeva O.I., Patiwael S., Dopler A., Hoefakker K., De Biasi S., Gibellini L., Mussini C., Guaraldi G. (2021). Identification and characterization of a SARS-CoV-2 specific CD8(+) T cell response with immunodominant features. Nat. Commun..

[6] Borella R., De Biasi S., Paolini A., Boraldi F., Lo Tartaro D., Mattioli M., Fidanza L., Neroni A., Caro-Maldonado A., Meschiari M. (2022). Metabolic reprograming shapes neutrophil functions in severe COVID-19. Eur. J. Immunol..

[7] Gibellini L., De Biasi S., Meschiari M., Gozzi L., Paolini A., Borella R., Mattioli M., Lo Tartaro D., Fidanza L., Neroni A. (2022). Plasma Cytokine Atlas Reveals the Importance of TH2 Polarization and Interferons in Predicting COVID-19 Severity and Survival. Front. Immunol..

[8] Osuchowski M.F., Winkler M.S., Skirecki T., Cajander S., Shankar-Hari M., Lachmann G., Monneret G., Venet F., Bauer M., Brunkhorst F.M. (2021). The COVID-19 puzzle: Deciphering pathophysiology and phenotypes of a new disease entity. Lancet Respir. Med..

[9] Wiech M., Chroscicki P., Swatler J., Stepnik D., De Biasi S., Hampel M., Brewinska-Olchowik M., Maliszewska A., Sklinda K., Durlik M. (2022). Remodeling of T Cell Dynamics During Long COVID Is Dependent on Severity of SARS-CoV-2 Infection. Front. Immunol..

[10] Lo Tartaro D., Neroni A., Paolini A., Borella R., Mattioli M., Fidanza L., Quong A., Petes C., Awong G., Douglas S. (2022). Molecular and cellular immune features of aged patients with severe COVID-19 pneumonia. Commun. Biol..

[11] Rydyznski Moderbacher C., Ramirez S.I., Dan J.M., Grifoni A., Hastie K.M., Weiskopf D., Belanger S., Abbott R.K., Kim C., Choi J. (2020). Antigen-Specific Adaptive Immunity to SARS-CoV-2 in Acute COVID-19 and Associations with Age and Disease Severity. Cell.

[12] Bertoletti A., T Tan A., Le Bert N. (2021). The T-cell response to SARS-CoV-2: Kinetic and quantitative aspects and the case for their protective role. Oxf. Open Immunol..

[13] Bange E.M., Han N.A., Wileyto P., Kim J.Y., Gouma S., Robinson J., Greenplate A.R., Hwee M.A., Porterfield F., Owoyemi O. (2021). CD8(+) T cells contribute to survival in patients with COVID-19 and hematologic cancer. Nat. Med..

[14] Sekine T., Perez-Potti A., Rivera-Ballesteros O., Stralin K., Gorin J.B., Olsson A., Llewellyn-Lacey S., Kamal H., Bogdanovic G., Muschiol S. (2020). Robust T Cell Immunity in Convalescent Individuals with Asymptomatic or Mild COVID-19. Cell.

[15] Le Bert N., Clapham H.E., Tan A.T., Chia W.N., Tham C.Y.L., Lim J.M., Kunasegaran K., Tan L.W.L., Dutertre C.A., Shankar N. (2021). Highly functional virus-specific cellular immune response in asymptomatic SARS-CoV-2 infection. J. Exp. Med..

[16] Reynolds C.J., Swadling L., Gibbons J.M., Pade C., Jensen M.P., Diniz M.O., Schmidt N.M., Butler D.K., Amin O.E., Bailey S.N.L. (2020). Discordant neutralizing antibody and T cell responses in asymptomatic and mild SARS-CoV-2 infection. Sci. Immunol..

[17] Bonifacius A., Tischer-Zimmermann S., Dragon A.C., Gussarow D., Vogel A., Krettek U., Godecke N., Yilmaz M., Kraft A.R.M., Hoeper M.M. (2021). COVID-19 immune signatures reveal stable antiviral T cell function despite declining humoral responses. Immunity.

[18] Grifoni A., Weiskopf D., Ramirez S.I., Mateus J., Dan J.M., Moderbacher C.R., Rawlings S.A., Sutherland A., Premkumar L., Jadi R.S. (2020). Targets of T Cell Responses to SARS-CoV-2 Coronavirus in Humans with COVID-19 Disease and Unexposed Individuals. Cell.

[19] Peng Y., Mentzer A.J., Liu G., Yao X., Yin Z., Dong D., Dejnirattisai W., Rostron T., Supasa P., Liu C. (2020). Broad and strong memory CD4(+) and CD8(+) T cells induced by SARS-CoV-2 in UK convalescent individuals following COVID-19. Nat. Immunol..

[20] Le Bert N., Tan A.T., Kunasegaran K., Tham C.Y.L., Hafezi M., Chia A., Chng M.H.Y., Lin M., Tan N., Linster M. (2020). SARS-CoV-2-specific T cell immunity in cases of COVID-19 and SARS, and uninfected controls. Nature.

[21] Nelde A., Bilich T., Heitmann J.S., Maringer Y., Salih H.R., Roerden M., Lubke M., Bauer J., Rieth J., Wacker M. (2021). SARS-CoV-2-derived peptides define heterologous and COVID-19-induced T cell recognition. Nat. Immunol..

[22] Pera A., Campos C., Corona A., Sanchez-Correa B., Tarazona R., Larbi A., Solana R. (2014). CMV latent infection improves CD8+ T response to SEB due to expansion of polyfunctional CD57+ cells in young individuals. PLoS ONE.

[23] Betts M.R., Nason M.C., West S.M., De Rosa S.C., Migueles S.A., Abraham J., Lederman M.M., Benito J.M., Goepfert P.A., Connors M. (2006). HIV nonprogressors preferentially maintain highly functional HIV-specific CD8+ T cells. Blood.

[24] Valverde-Villegas J.M., Matte M.C., de Medeiros R.M., Chies J.A. (2015). New Insights about Treg and Th17 Cells in HIV Infection and Disease Progression. J. Immunol. Res..

[25] Zambrano-Zaragoza J.F., Romo-Martinez E.J., Duran-Avelar Mde J., Garcia-Magallanes N., Vibanco-Perez N. (2014). Th17 cells in autoimmune and infectious diseases. Int. J. Inflamm..

[26] Frank K., Paust S. (2020). Dynamic Natural Killer Cell and T Cell Responses to Influenza Infection. Front. Cell. Infect. Microbiol..

[27] Chen X., Liu S., Goraya M.U., Maarouf M., Huang S., Chen J.L. (2018). Host Immune Response to Influenza A Virus Infection. Front. Immunol..

[28] Hornick E.E., Zacharias Z.R., Legge K.L. (2019). Kinetics and Phenotype of the CD4 T Cell Response to Influenza Virus Infections. Front. Immunol..

[29] Damsker J.M., Hansen A.M., Caspi R.R. (2010). Th1 and Th17 cells: Adversaries and collaborators. Ann. N. Y. Acad. Sci..

[30] Betts M.R., Koup R.A. (2004). Detection of T-cell degranulation: CD107a and b. Methods Cell Biol..

[31] Schmidt M.E., Varga S.M. (2018). The CD8 T Cell Response to Respiratory Virus Infections. Front. Immunol..

[32] Meckiff B.J., Ladell K., McLaren J.E., Ryan G.B., Leese A.M., James E.A., Price D.A., Long H.M. (2019). Primary EBV Infection Induces an Acute Wave of Activated Antigen-Specific Cytotoxic CD4^+^ T Cells. J. Immunol..

[33] Casazza J.P., Betts M.R., Price D.A., Precopio M.L., Ruff L.E., Brenchley J.M., Hill B.J., Roederer M., Douek D.C., Koup R.A. (2006). Acquisition of direct antiviral effector functions by CMV-specific CD4^+^ T lymphocytes with cellular maturation. J. Exp. Med..

[34] Nemes E., Bertoncelli L., Lugli E., Pinti M., Nasi M., Manzini L., Manzini S., Prati F., Borghi V., Cossarizza A. (2010). Cytotoxic granule release dominates gag-specific CD4+ T-cell response in different phases of HIV infection. Aids.

[35] Betts M.R., Brenchley J.M., Price D.A., De Rosa S.C., Douek D.C., Roederer M., Koup R.A. (2003). Sensitive and viable identification of antigen-specific CD8+ T cells by a flow cytometric assay for degranulation. J. Immunol. Methods.

[36] Tan A.T., Linster M., Tan C.W., Le Bert N., Chia W.N., Kunasegaran K., Zhuang Y., Tham C.Y.L., Chia A., Smith G.J.D. (2021). Early induction of functional SARS-CoV-2-specific T cells associates with rapid viral clearance and mild disease in COVID-19 patients. Cell Rep..

[37] Moss P. (2022). The T cell immune response against SARS-CoV-2. Nat. Immunol..

[38] Diao B., Wang C., Tan Y., Chen X., Liu Y., Ning L., Chen L., Li M., Liu Y., Wang G. (2020). Reduction and Functional Exhaustion of T Cells in Patients With Coronavirus Disease 2019 (COVID-19). Front. Immunol..

[39] Snell L.M., Osokine I., Yamada D.H., De la Fuente J.R., Elsaesser H.J., Brooks D.G. (2016). Overcoming CD4 Th1 Cell Fate Restrictions to Sustain Antiviral CD8 T Cells and Control Persistent Virus Infection. Cell Rep..

[40] Ferretti A.P., Kula T., Wang Y., Nguyen D.M.V., Weinheimer A., Dunlap G.S., Xu Q., Nabilsi N., Perullo C.R., Cristofaro A.W. (2020). Unbiased Screens Show CD8(+) T Cells of COVID-19 Patients Recognize Shared Epitopes in SARS-CoV-2 that Largely Reside outside the Spike Protein. Immunity.

[41] Reuter M.A., Pombo C., Betts M.R. (2012). Cytokine production and dysregulation in HIV pathogenesis: Lessons for development of therapeutics and vaccines. Cytokine Growth Factor Rev..

[42] Carvalho-Gomes A., Cubells A., Pallares C., Corpas-Burgos F., Berenguer M., Aguilera V., Lopez-Labrador F.X. (2022). Cytomegalovirus specific polyfunctional T-cell responses expressing CD107a predict control of CMV infection after liver transplantation. Cell. Immunol..

[43] Pan K., Chiu Y., Huang E., Chen M., Wang J., Lai I., Singh S., Shaw R.M., MacCoss M.J., Yee C. (2021). Mass spectrometric identification of immunogenic SARS-CoV-2 epitopes and cognate TCRs. Proc. Natl. Acad. Sci. USA.

[44] Troyano-Hernáez P., Reinosa R., Holguín Á. (2021). Evolution of SARS-CoV-2 Envelope, Membrane, Nucleocapsid, and Spike Structural Proteins from the Beginning of the Pandemic to September 2020: A Global and Regional Approach by Epidemiological Week. Viruses.

[45] WHO Working Group on the Clinical Characterisation and Management of COVID-19 infection (2020). A minimal common outcome measure set for COVID-19 clinical research. Lancet Infect. Dis..

[46] Cossarizza A., Chang H.D., Radbruch A., Abrignani S., Addo R., Akdis M., Andra I., Andreata F., Annunziato F., Arranz E. (2021). Guidelines for the use of flow cytometry and cell sorting in immunological studies (third edition). Eur. J. Immunol..

[47] De Biasi S., Tartaro D.L., Gibellini L., Paolini A., Quong A., Petes C., Awong G., Douglas S., Lin D., Nieto J. (2021). Endogenous control of inflammation characterizes pregnant women with asymptomatic or paucisymptomatic SARS-CoV-2 infection. Nat. Commun..

[48] Roederer M., Nozzi J.L., Nason M.C. (2011). SPICE: Exploration and analysis of post-cytometric complex multivariate datasets. Cytometry Part A.

