# Supplementary material for: Patients Recovering from Severe COVID-19 Develop a Polyfunctional Antigen-Specific CD4+ T Cell Response
Source: Int J Mol Sci. 2022 Jul 20;23(14):8004. doi: 10.3390/ijms23148004 (PMC9323836; doi:10.3390/ijms23148004)
Supplement: Supplementary file 1 [file ijms-23-08004-s001.zip › ijms-1820271-supplementary.pptx]

## Slide 1
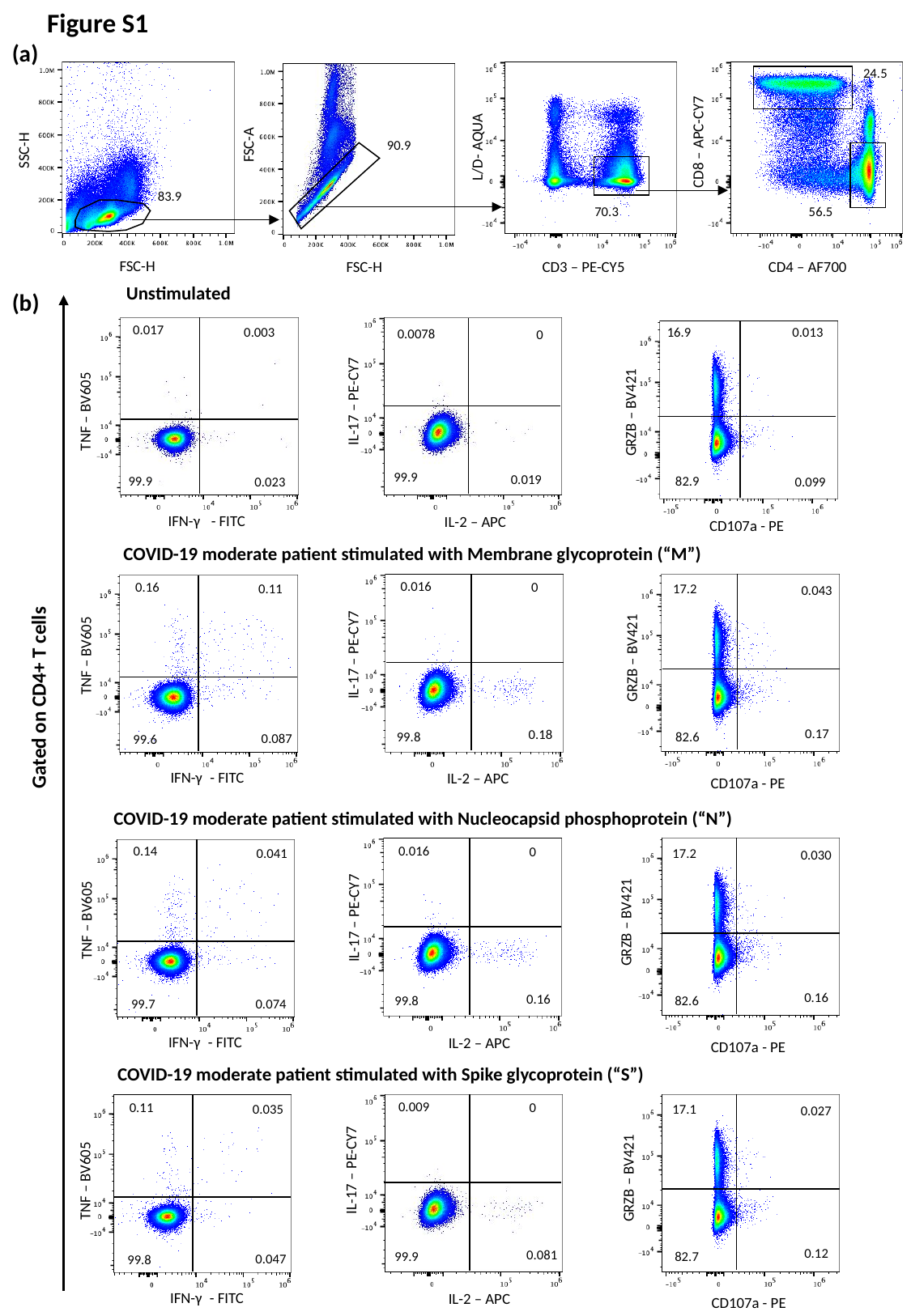

Figure S1
(a)
24.5
CD8 – APC-CY7
FSC-A
L/D- AQUA
SSC-H
90.9
83.9
70.3
56.5
FSC-H
CD3 – PE-CY5
CD4 – AF700
FSC-H
Unstimulated
(b)
16.9
0.013
GRZB – BV421
82.9
0.099
0.017
0.003
0.0078
0
IL-17 – PE-CY7
TNF – BV605
99.9
0.019
99.9
0.023
IFN-γ - FITC
IL-2 – APC
CD107a - PE
COVID-19 moderate patient stimulated with Membrane glycoprotein (“M”)
0.016
0.16
0
17.2
0.11
0.043
Gated on CD4+ T cells
TNF – BV605
IL-17 – PE-CY7
GRZB – BV421
0.17
0.18
82.6
99.8
0.087
99.6
IFN-γ - FITC
IL-2 – APC
CD107a - PE
COVID-19 moderate patient stimulated with Nucleocapsid phosphoprotein (“N”)
0.016
0.14
0
17.2
0.041
0.030
TNF – BV605
IL-17 – PE-CY7
GRZB – BV421
0.16
0.16
82.6
99.8
0.074
99.7
IFN-γ - FITC
IL-2 – APC
CD107a - PE
COVID-19 moderate patient stimulated with Spike glycoprotein (“S”)
0.009
0.11
0
17.1
0.035
0.027
IL-17 – PE-CY7
TNF – BV605
GRZB – BV421
0.12
0.081
82.7
99.9
0.047
99.8
IFN-γ - FITC
IL-2 – APC
CD107a - PE

## Slide 2
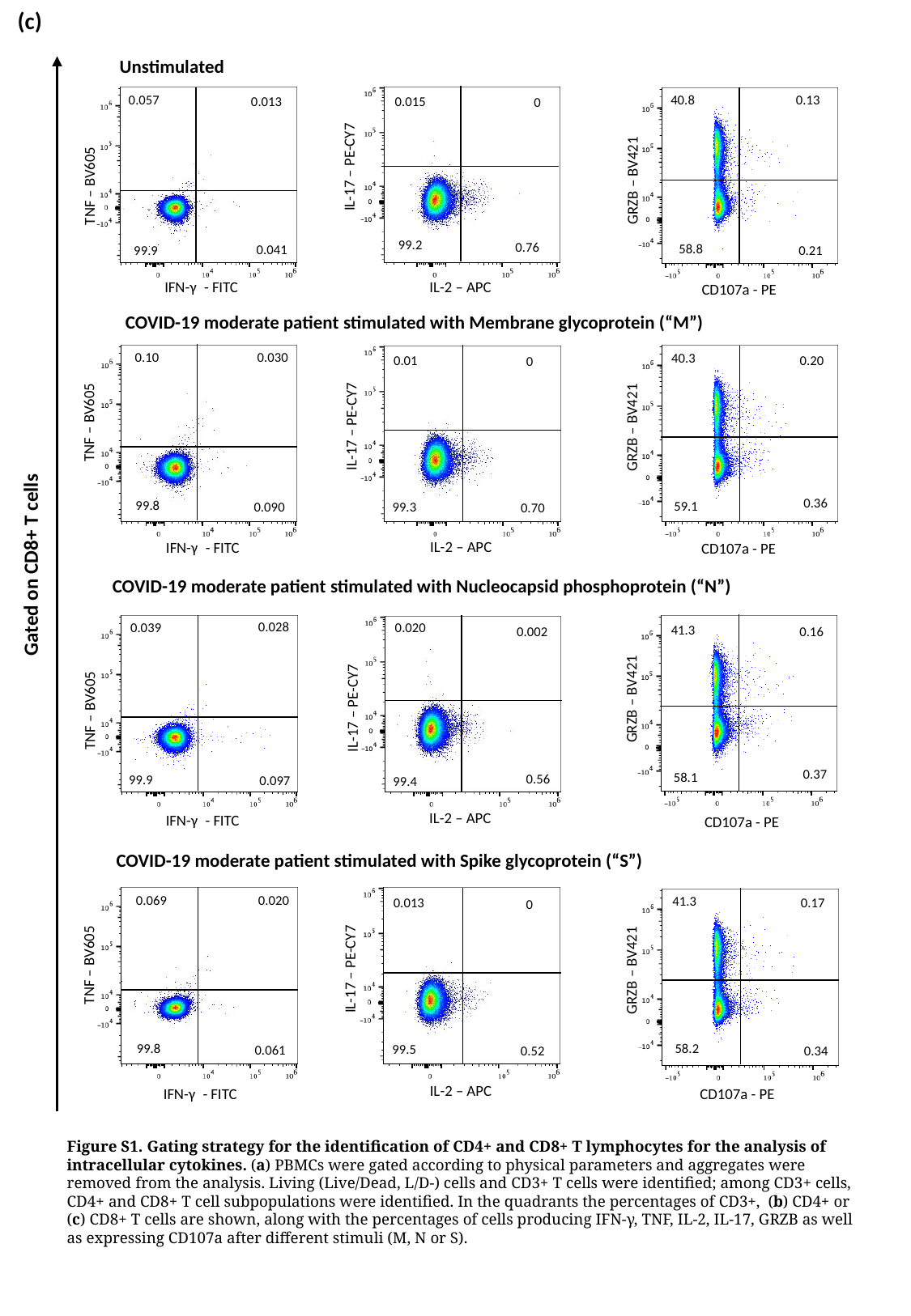

(c)
Unstimulated
40.8
0.057
0.13
0.015
0.013
0
IL-17 – PE-CY7
GRZB – BV421
TNF – BV605
99.2
0.76
58.8
0.041
0.21
99.9
IL-2 – APC
IFN-γ - FITC
CD107a - PE
COVID-19 moderate patient stimulated with Membrane glycoprotein (“M”)
0.10
0.030
40.3
0.20
0.01
0
TNF – BV605
IL-17 – PE-CY7
GRZB – BV421
Gated on CD8+ T cells
0.36
99.8
59.1
99.3
0.090
0.70
IL-2 – APC
IFN-γ - FITC
CD107a - PE
COVID-19 moderate patient stimulated with Nucleocapsid phosphoprotein (“N”)
0.028
0.039
0.020
41.3
0.002
0.16
GRZB – BV421
TNF – BV605
IL-17 – PE-CY7
0.37
58.1
0.56
99.9
0.097
99.4
IL-2 – APC
IFN-γ - FITC
CD107a - PE
COVID-19 moderate patient stimulated with Spike glycoprotein (“S”)
0.069
0.020
41.3
0.17
0.013
0
TNF – BV605
IL-17 – PE-CY7
GRZB – BV421
99.8
58.2
99.5
0.061
0.52
0.34
IL-2 – APC
CD107a - PE
IFN-γ - FITC
Figure S1. Gating strategy for the identification of CD4+ and CD8+ T lymphocytes for the analysis of intracellular cytokines. (a) PBMCs were gated according to physical parameters and aggregates were removed from the analysis. Living (Live/Dead, L/D-) cells and CD3+ T cells were identified; among CD3+ cells, CD4+ and CD8+ T cell subpopulations were identified. In the quadrants the percentages of CD3+, (b) CD4+ or (c) CD8+ T cells are shown, along with the percentages of cells producing IFN-γ, TNF, IL-2, IL-17, GRZB as well as expressing CD107a after different stimuli (M, N or S).

## Slide 3
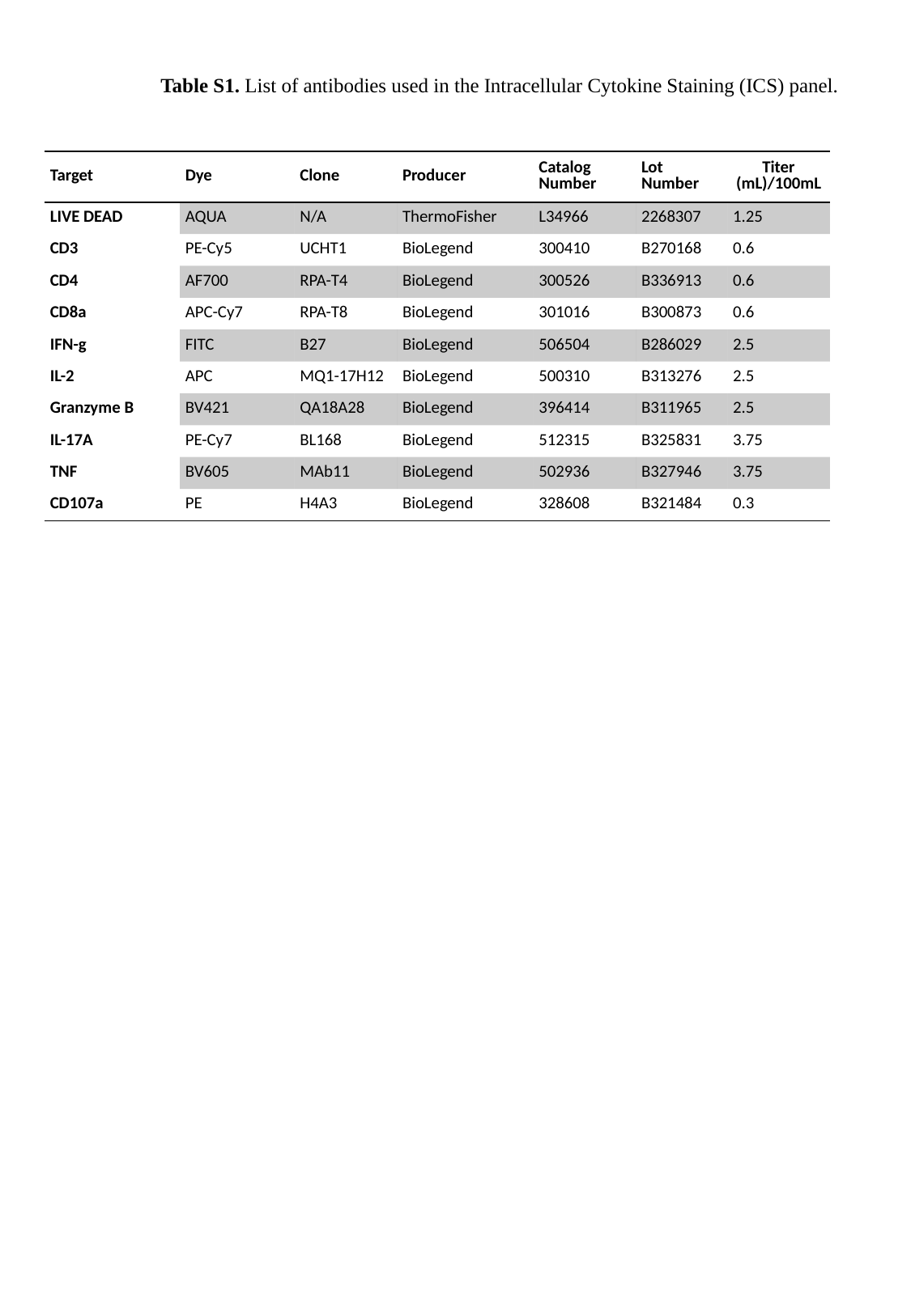

Table S1. List of antibodies used in the Intracellular Cytokine Staining (ICS) panel.
| Target | Dye | Clone | Producer | Catalog Number | Lot Number | Titer (mL)/100mL |
| --- | --- | --- | --- | --- | --- | --- |
| LIVE DEAD | AQUA | N/A | ThermoFisher | L34966 | 2268307 | 1.25 |
| CD3 | PE-Cy5 | UCHT1 | BioLegend | 300410 | B270168 | 0.6 |
| CD4 | AF700 | RPA-T4 | BioLegend | 300526 | B336913 | 0.6 |
| CD8a | APC-Cy7 | RPA-T8 | BioLegend | 301016 | B300873 | 0.6 |
| IFN-g | FITC | B27 | BioLegend | 506504 | B286029 | 2.5 |
| IL-2 | APC | MQ1-17H12 | BioLegend | 500310 | B313276 | 2.5 |
| Granzyme B | BV421 | QA18A28 | BioLegend | 396414 | B311965 | 2.5 |
| IL-17A | PE-Cy7 | BL168 | BioLegend | 512315 | B325831 | 3.75 |
| TNF | BV605 | MAb11 | BioLegend | 502936 | B327946 | 3.75 |
| CD107a | PE | H4A3 | BioLegend | 328608 | B321484 | 0.3 |
